# Low-Pressure H_2_, NH_3_ Microwave Plasma Treatment of Polytetrafluoroethylene (PTFE) Powders: Chemical, Thermal and Wettability Analysis

**DOI:** 10.3390/ma8052258

**Published:** 2015-04-28

**Authors:** Harald Hunke, Navneet Soin, Tahir H. Shah, Erich Kramer, Alfons Pascual, Mallampalli Sri Lakshmi Karuna, Elias Siores

**Affiliations:** 1 Institute for Materials Research & Innovation (IMRI), University of Bolton, Deane Road, Bolton BL3 5AB, UK; E-Mails: haraldhunke@hotmail.com (H.H.); ths1@bolton.ac.uk (T.H.S.); es3@bolton.ac.uk (E.S.); 2 Institute of Polymer Engineering, University of Applied Sciences (UAS), Northwestern Switzerland, 5210 Windisch, Switzerland; E-Mails: erich.kramer@fhnw.ch (E.K.); alfons.pascual@fhnw.ch (A.P.); 3 Center for Lipid Research, Indian Institute of Chemical Technology (IICT), Hyderabad 500007, India; E-Mail: mslkaruna@gmail.com

**Keywords:** polytetrafluoroethylene (PTFE), low-pressure microwave plasma, ESCA/XPS, degeneration, thermal stability

## Abstract

Functionalization of Polytetrafluoroethylene (PTFE) powders of ~6 μm particle size is carried out using low-pressure 2.45 GHz H_2_, NH_3_ microwave plasmas for various durations (2.5, 10 h) to chemically modify their surface and alter their surface energy. The X-ray Photoelectron Spectroscopy (XPS) analyses reveal that plasma treatment leads to significant defluorination (F/C atomic ratio of 1.13 and 1.30 for 10 h NH_3_ and H_2_ plasma treatments, respectively *vs.* 1.86 for pristine PTFE), along with the incorporation of functional polar moieties on the surface, resulting in enhanced wettability. Analysis of temperature dependent XPS revealed a loss of surface moieties above 200 °C, however, the functional groups are not completely removable even at higher temperatures (>300 °C), thus enabling the use of plasma treated PTFE powders as potential tribological fillers in high temperature engineering polymers. Ageing studies carried over a period of 12 months revealed that while the surface changes degenerate over time, again, they are not completely reversible. These functionalised PTFE powders can be further used for applications into smart, high performance materials such as tribological fillers for engineering polymers and bio-medical, bio-material applications.

## 1. Introduction

Polytetrafluoroethylene (PTFE) offers outstanding properties such as high chemical inertness, heat resistance and excellent dielectric properties. Owing to its low coefficient of friction, PTFE is widely used as a sintered pure polymer, a reinforcement polymer and as an additive in polymer compounds to improve the friction and wear properties [1,2,3,4,5,6,7,8]. However, in some cases, its applications are hampered due to PTFE’s inherent poor chemical reactivity, low wettability and poor adhesion with other materials [3]. Hence, a considerable amount of effort has been undertaken to alter the chemical structure of PTFE and improve the surface activity by means of chemical etching (for ex. reduction with sodium naphthalene), electron beam irradiation or treatment with various plasma techniques [1,2,4,5,6,7,8,9].

The use of plasma techniques for the treatment of polymers is a versatile method to create novel surface functionalities by incorporating surface functional groups without altering the bulk properties. Plasma treatment is an environmentally friendly technique, as it does not expose toxic organic components to the environment. Moreover, plasma treatments can be performed at room temperature, with the process being easy to control and relatively low cost [10]. Yasuda *et al.* showed that various functional groups can be incorporated on PTFE films by plasma treatment by varying process gases such as argon, nitrogen, ammonia, or hydrogen [11]. From a technological point of view, the plasma treatment of powders is difficult owing to the three-dimensional geometry of the particles, need for sophisticated mixing techniques to overcome the aggregation phenomenon and the large surface area of the powders which are to be modified [10]. However, nearly 60% of all the products and intermediates in the chemical, pharmaceutical or food industry are handled or processed in the form of powders only. The high surface to volume ratio and macroscopic behavior of the powder mainly depends on the surface chemistry and structure of the individual powder particles, and hence it is of prime importance to develop processes that can impart functionality and modification at the particle scale while keeping the bulk properties unaffected. Due to its low coefficient of friction, PTFE powders are often used as tribological filler for reducing the static and dynamic friction, as well as wear rate of engineering polymers such as polyethersulfone, polyamide, *etc.* However, as mentioned earlier, the huge differences in surface energy, inherent low chemical reactivity and poor interfacial adhesion of PTFE with other polymers hampers its applications. For high performance compounds, it is important to develop processes which can impart surface functionality to PTFE powders to enhance their dispersion in the thermoplastic materials while maintaining the excellent frictional properties. Moreover, the enhanced interaction between the plasma functionalised PTFE and the host polymer matrix due to the polar functional groups, allows enhanced force absorption capabilities, lower coefficient of friction and wear rates as compared to the pristine PTFE compounds [12]. The incorporation of functional groups reduce the hydrophobic and oleophobic properties of the PTFE preventing it from agglomeration; thereby promoting homogeneous compounding with other polymeric materials such as polyphenylensulfide, polyethersulfone or polyamide [13,14]. To this effect, several concepts of plasma reactors for polymer powders have been developed, including plasma downer reactors, plasma-fluidized bed reactors or plasma batch reactors, all of which have shown varying degree of success [15,16,17]. Many publications on the plasma treatment of PTFE films, PTFE sheets or sintered PTFE specimens have been reported, and the plasma treatment of polymer powders such as polypropylene, polyethylene and ultrahigh molecular weight polyethylene, polyamide, and polyoxymethylene has been evaluated [18,19,20,21,22,23]. However, to the best of our knowledge, studies on the plasma treatment of PTFE powders have not been reported as yet.

In the present work, a low-pressure microwave (MW) plasma batch reactor was used to treat polytetrafluoroethylene (PTFE) powders with H_2_ and NH_3_ as process gases. The effects of plasma treatment time were studied using X-ray Photoelectron Spectroscopy (XPS), Fourier transform Infrared Spectroscopy (FTIR), Differential Scanning Calorimetry (DSC) and Washburne wettability measurements. The effect of long term ambient storage of H_2_ plasma treated powders was also studied to understand the plasma degeneration effects. Detailed temperature-dependent XPS analysis of the samples was also carried out to ascertain the thermal stability of the functional groups on PTFE surfaces. Furthermore, we have investigated the feasibility of scaling up of the plasma treatment process by performing preliminary analysis on a scaled-up reactor with a modified drum of 25 kg capacity.

## 2. Results and Discussion

In the literature, the effects of NH_3_ and H_2_ plasmas on PTFE films have been studied extensively, and XPS results have demonstrated that these treatments lead to partial loss of fluorine (defluorination) accompanied by the production of hydrocarbons, cross-linking, chain scission and, depending on the feed gas used, incorporation of oxygen- and nitrogen-containing groups [5,21,24]. All the PTFE powder samples (~3 kg quantity) were treated at a working pressure of 0.8 mbar and 270 W power for a duration of 2.5 and 10 h for both H_2_ and NH_3_ plasmas, except for scale-up process (25 kg quantity) which was treated for 900 W at 0.4 mbar pressure for 10 h with H_2_ plasma only. As shown in Table 1, both the H_2_ and NH_3_ plasma treatments were successful in altering the surface of the PTFE with varying levels of defluorination. In fact, the lowest fluorine to carbon atomic ratio was observed on the sample treated for ten hours with NH_3_ as a process gas (PTFE NH_3_/10 h; F/C ratio: 1.13), followed by the sample treated for ten hours with H_2_ (PTFE H_2_/10 h/2012; ratio: 1.30) as compared to pristine PTFE (F/C ratio 1.86). The effects of variation of plasma gas and treatment time are discussed in detail in the following sections.

**Table 1:** Atomic percentages and associated elemental ratios of PTFE samples.

Sample	Atomic Percentage [%]				Elemental Ratios		
	C	F	O	N	F/C	O/C	N/C
Pristine PTFE	34.94	65.06	--	--	1.86	--	--
PTFE NH_3_/2.5 h	39.48	58.35	0.92	1.24	1.48	0.02	0.03
PTFE NH_3_/10 h	44.85	50.81	1.66	2.68	1.13	0.04	0.06
PTFE H_2_/10 h/2011	36.74	62.20	0.45	0.61	1.69	0.01	0.02
PTFE H_2_/10 h/2012	43.11	56.04	0.85	--	1.30	0.02	0.00
PTFE Max H_2_/10 h	36.21	63.72	0.07	--	1.76	0.002	0.00

### 2.1. X-ray Photoelectron Spectroscopy

#### 2.1.1. Pristine PTFE Powders

**Figure 1:**
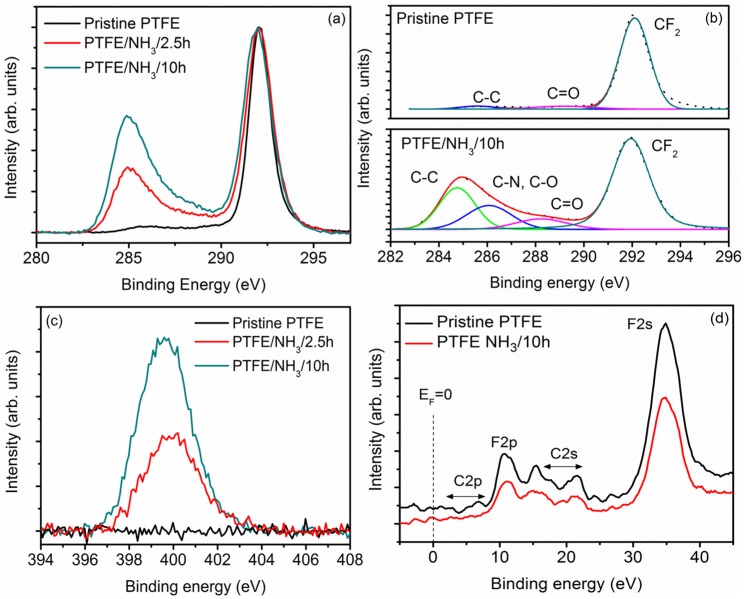
(**a**) High resolution C1s spectra of pristine and NH_3_ plasma treated PTFE powders. (**b**) Deconvolution of C1s spectra of pristine and PTFE/NH_3_/10 h samples. (**c**) Increase in the N1s signal intensity as a function of NH_3_ plasma treatment time. (**d**) XPS valence band spectra of pristine and PTFE/NH_3_/10 h samples.

Figure 1a,b shows the high-resolution C1s spectrum for the pristine PTFE powder samples. The core-level peak is quite symmetrical with a narrow full width at half-maxima (FWHM) of 1.45 eV and the binding energy (292.2 eV) consistent with the (-CF_2_-)_n_ coordination. A broad discernible band at approx. 285 eV, ascribed to adventitious –C-H_x_- moieties is also observed [21,24]. The binding energy difference between the (–CF_2_-)_n_ and the –C-H_x_- peaks of nearly 6.6 eV is quite similar to the values reported earlier in literature (6.5 eV) [25]. The high resolution F1s spectrum (Suppl. Figure S1) at 689 eV presented a FWHM of 1.92 eV and was quite symmetrical in nature [21,24,25]. As shown in Table 1, the measured value of fluorine to carbon (F/C) ratio of 1.86, matched well with the values reported in the literature [21,24,25]. It must be noted that for pristine samples, the O1s signal was quite weak and no significant quantities of oxygen could be detected on the pristine PTFE surfaces.

#### 2.1.2. NH3 Plasma-Treated Powders

The surface composition of NH_3_ plasma treated PTFE powder samples, measured by XPS, is shown in Table 1. As compared to pristine PTFE, significant changes can be observed in the C1s envelope of NH_3_ plasma treated samples, especially with regards to the increase in the peak intensities around 285 eV (Figure 1a,b). In fact, an increase in the asymmetry and the width of the (-CF_2_-)_n_ peak with the increase in the treatment time could also be observed clearly. The increase in the component at 285 eV has been attributed to the formation of –C–C–, -C=C- and –C–H_x_ moieties, although these groups cannot be resolved separately; while, the peaks located at 286.0, 288.2 and 291.9 eV can be ascribed to C-O/C-N and C=O, respectively [25]. It should be noted that the C-C bonds arise due to the strong surface defluorination and chain scission of the PTFE and have been reported earlier as well [26]. In view of the extensive loss of fluorine (65 at.% in pristine *vs.* 50.8 at.% for NH_3_ treated samples), Wilson *et al.* have ascribed the peaks in this region to –C-CF and –C-F formed by the reaction of CF_2_ radical with liberated fluorine and oxygen in the plasma chamber [21,24]. For the NH_3_ treated samples, the core F1s level increased in asymmetry and the FWHM increased to 2.25 eV *vs.* 1.92 eV for pristine samples (Suppl. Figure S1). In fact, with the increase in the treatment time from 2.5 h to 10 h, an incremental increase in defluorination was observed with the F/C ratio going down from 1.48 (for 2.5 h treatment time) to 1.13 (for 10 h treatment time) as compared to 1.86 for pristine samples. The defluorination effect itself is attributed to the physical sputtering of the PTFE surface upon nitrogen ion bombardment in plasma (Suppl. Figure S2). Accordingly, the N content increased from 1.24 to 2.68 at.% along with an increase in the O content from 0.92 to 1.66 at.%, for 2.5 h and 10 h samples, respectively. This oxygen, which was detected at the surface after NH_3_ plasma treatment, can occur due to O-grafting during or after the treatment. The incorporation of oxygen upon polymer surfaces in non-oxygen plasma treatments is quite common and occurs due to presence of free radicals that are created on a polymer surface during the plasma treatment. Upon exposure to the ambient environment, these unreacted free radicals interact with oxygen to create the oxygen functionalities on the polymer surface [21,24]. In addition, the possibility of leakage of air into the plasma chamber during plasma processing can lead to the formation of oxygen moieties. As the leak rate of the chamber was quite low, we believe that the oxygen moieties were formed upon the exposure of the powder samples to the ambient environment only. However, further systematic studies are required to ascertain the effect of exposure time on the increase in the oxygen content, such as those performed by Werner *et al.* [26] and Wilson *et al.* [27]. In Wilson’s, it was observed that plasma treated PTFE sheets undergo further chemical changes as a function of time with the reaction kinetics dependent on the storage media as well as the gas used for plasma treatment [27]. Especially for samples stored in air, a steady state was reached within one month with the reaction attributed to the replacement of amines by either hydroxyl or amide groups [27]. Even though the increase in the treatment time nearly doubled the N content, the N1s core level signal did not show any changes in its shape, implying the nature of moieties attached to the surface remained similar (Figure 1c). The N1s signal was deconvoluted into two major components at 399.5 eV and 401.2 eV, corresponding to C-N and N-C-O configurations, respectively, with C-N being the dominant component [21,24,25]. The presence of these functional polar groups on PTFE molecule can lower its surface energy and improve the wettability. The valence-band spectrum for pristine and NH_3_ treated PTFE is shown in Figure 1d. The two major peaks associated with C-F bonding appear at approx. 11 eV and 35 eV, corresponding to F2p and F2s, respectively [21]. The C2p shows up as low intensity peak between 5 eV to 12 eV and is indicative of C-H bonding [21]. The C2s peak gives rise to signals at 16 eV and 22 eV corresponding to C-C anti-bonding and bonding orbitals [22]. Upon NH_3_ plasma treatment, a decrease in the intensity of F2p and F2s signals was observed along with a broadening of the C2p signal, representative of the formation of C-N groups upon NH_3_ plasma treatment [21]. These results are in confirmation of the results previously reported on the NH_3_ plasma treatment of PTFE sheets by various groups [21,24].

#### 2.1.3. H2 Plasma-Treated Powders

**Figure 2:**
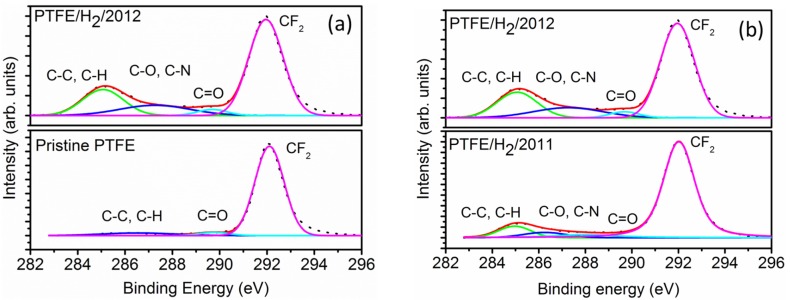
(**a**) High resolution C1s spectra of pristine and H_2_ plasma treated PTFE powders showing the efficacy of the process. (**b**) High resolution C1s spectra of PTFE/H_2_/2011 and PTFE/H_2_/2012 samples showing the degeneration of plasma treated surfaces.

The surface composition of H_2_ plasma treated PTFE powder samples, as measured by XPS, is shown in Table 1 and it can be clearly observed that the treatment led to the attachment of mainly hydrogen and oxygen moieties. For PTFE H_2_/10 h/2012 samples, the F/C ratio is 1.30 which is much lower than that of pristine PTFE (F/C ratio of 1.86), accompanied by an increase in the oxygen content to 0.85 at.% (O/C ratio of 0.02 *vs.* no oxygen detected on pristine PTFE surfaces), indicative of the defluorination of PTFE powders by H_2_ plasma treatment. This small increase in the O/C ratio to 0.02 upon plasma treatment means that some oxygen functional groups were formed simultaneously during/after the defluorination process. These oxygen functional groups correspond to the components at 287.7 and 290.0 eV, which are assigned to C-O and C=O bonds, respectively (Figure 2a). Inagaki *et al.* have ascribed these peak positions in H_2_ treated PTFE surfaces to O-CH-CF_n_, CHF and O=C-CF_n_, CHF-CF_n_ groups, respectively [2]. The defluorination mechanism in H_2_ plasma treated powders can be explained using the following schematic. The formation of H• radicals in the H_2_ plasma abstract fluorine atoms from the PTFE surface to form free carbon radicals [5]. These carbon radicals recombine with other hydrogen radicals in the hydrogen plasma to form C-H bonds which correspond to the defluorination mechanism [5]. However, similar to the NH_3_ plasma treatment, not all the carbon radicals which are formed by hydrogen abstraction recombine with the other radicals. A part of these carbon radicals remain on the surface of the PTFE particles even after the plasma processing has been completed. When the chamber is opened and the powders exposed to the ambient environment, the remaining radicals come in contact with the air to form peroxide groups which then further modify into hydroxyl and carbonyl groups observed in the XPS analysis. The samples treated with H_2_ plasma, PTFE H_2_/10 h/2012 and PTFE H_2_/10 h/2011 showed a small peak at 294 eV, which according to Vandencasteele, can be attributed to CF_3_ components produced via chain scission [28].

#### 2.1.4. Thermal Stability of Functional Groups for H2, NH3 Plasma Treated PTFE

**Figure 3:**
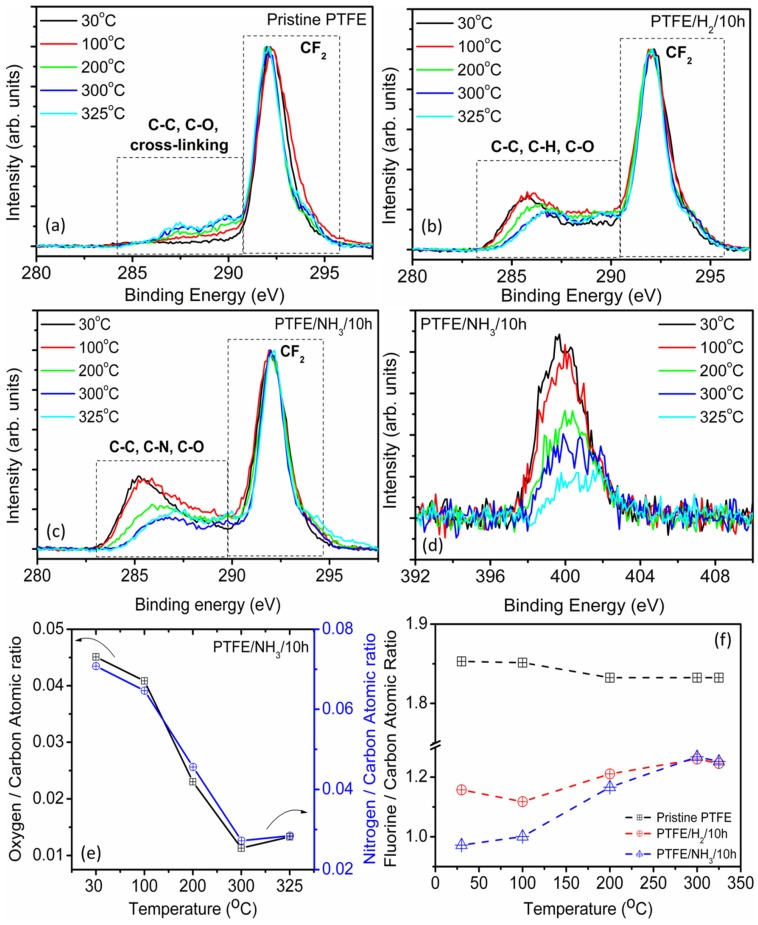
Temperature dependent XPS (**a**) C1s core level XPS spectra for pristine PTFE. (**b**) C1s core level XPS spectra for PTFE/H2/10 h sample. (**c**) C1s core level XPS spectra for PTFE/NH3/10 h sample. (**d**) N1s core level XPS spectra showing the decrease in the signal intensity. (**e**) Changes in the O/C and N/C atomic ratios for PTFE/NH3/10 h sample as function of temperature. (**f**) Evolution of F/C atomic ratio for pristine and plasma treated PTFE as a function of temperature.

**Figure 4:**
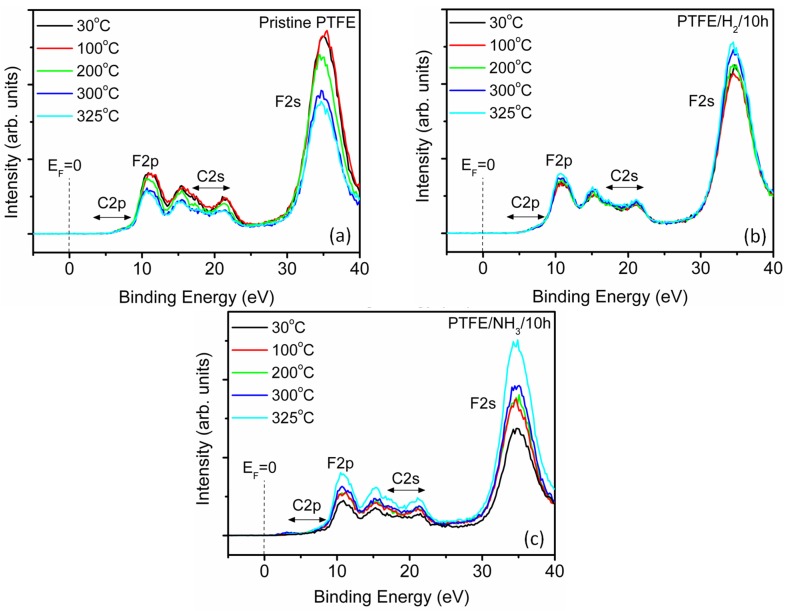
Evolution of valence band as a function of temperature for (**a**) Pristine PTFE. (**b**) H_2_ plasma treated PTFE. (**c**) NH_3_ plasma treated PTFE.

For potential use as tribological lubricants in plastic compounds, the PTFE powders are often further subjected to processing with other polymeric materials using techniques such as twin screw melt compounding during which the PTFE is subjected to high temperature conditions near/above its melting temperatures. As the polar groups on PTFE surfaces are mobile and their adhesion to the surface low, the high temperature behavior and stability of the polar groups needs to be ascertained and investigated further [26,27,29]. To this effect, temperature dependent XPS analysis of PTFE powders was carried out as per the procedure discussed in the experimental section. For pristine PTFE samples, the heating of samples up till 200 °C, (Figure 3a) did not produce any appreciable change in the C1s core level XPS spectrum (Suppl. Table T1). However, further heating of the sample to 300 °C and beyond led to the appearance of peaks near 287.5 and 289 eV, attributed to the cross-linking in the polymer, which may occur due to either high temperature effects or as a result of X-ray exposure [30]. From the literature, it is known that PTFE is highly susceptible to X-ray damage and a combination of high temperature heating in conjunction with the X-ray exposure could have led to the cross-linking of the PTFE polymer samples. It should be noted that the peaks at ~289 eV, corresponding to the cross-linking become more prominent at temperatures above 200 °C. For plasma functionalised PTFE powders, the changes in the C1s core level spectrum are much more pronounced (see Figure 3b,c). In fact, for PTFE/NH_3_/10 h samples, the C1s core level signal showed a continuous reduction in the intensity of bands in the region 285–289 eV, ascribed earlier (see Section 2.1) to C-C, C-N and C-O groups, indicating at the removal of nitrogen functionalities with increase in temperature, accompanied by a concurrent reduction in the intensity of the N1s signal (Figure 3d). As discussed earlier in Section 2.1, the attachment of nitrogen moieties to the PTFE surface is usually accompanied by introduction of oxygen moieties to the surface as well. Incidentally, the behavior of loss of nitrogen functionality (N/C atomic ratio) is followed closely by the loss of oxygen moieties (O/C atomic ratio) as seen in Figure 3e. For pristine PTFE samples, the F/C ratio did not vary significantly as a function of temperature; however, for both H_2_ and NH_3_ plasma treated PTFE surfaces, the F/C ratio followed a very similar trend indicating a similar process for the removal of hydrogen and nitrogen functional groups from the surface. Nevertheless, in spite of the high temperatures used during the measurement, complete removal of the polar species for both H_2_ and NH_3_ plasma treated samples was not observed and the difference between the F/C ratios of pristine (~1.85) and plasma treated PTFE samples (~1.26) was significant, indicative of the suitability of the plasma modification route for producing relatively stable moieties on the PTFE surfaces (Figure 3f). The valence-band spectrum for pristine PTFE is shown in Figure 4a with major peaks corresponding to F2p and F2s appearing at 11 and 35 eV, respectively. With the increase in the temperature, the overall intensity of the valence band signal reduced, with a corresponding reduction in the F2s band (35 eV) intensity which may be attributed to the cross-linking of PTFE [25]. For PTFE/H_2_ samples, with an increase in the temperature, the F2s signal appeared to increase in intensity which could be related to the removal of hydrogenated species from the PTFE surface and associated recovery of -CF_2_- backbone (Figure 4b, Suppl. Table T1). Similar trend was observed for PTFE/NH_3_ samples, where an increase in the F2s signal was observed (Figure 4c, Suppl. Table T1) in conjunction with the restoration of fine structure in the region 15–20 eV was observed, which could be related to the removal of nitrogenated species [27].

### 2.2. Effects of Ageing on H2 Plasma Treated PTFE

The ageing effects of plasma-treated polymers have been investigated by several groups and it has been shown that the chemical changes induced by plasma treatment are partially or completely reversible as a function of time [24]. Wilson *et al.* have studied the ageing effects of short plasma treatment of PTFE sheets and found that the plasma treatment was partially or totally reversible after storage [27]. In their study, the storage time was one month, however, in our case, we have envisaged to study the long-term effects of plasma treated PTFE powders. Two principal mechanisms, namely chain relaxation process and further chemical reactions with the storage environment have been established to explain the ageing effects of plasma treated polymers [24]. To study the effects of H_2_ plasma treated samples, a sample which was treated (PTFE H_2_/10 h/2011) under same plasma conditions as that of PTFE H_2_/10 h/2012, was stored at room temperature conditions and ambient environment. The sample PTFE H_2_/10 h/2011, which was stored for 12 months, exhibited a higher F/C ratio (1.69) than PTFE H_2_/10 h/2012 (1.13), but a lower value than that of pristine PTFE (1.86) (Figure 2b). In fact, for the aged sample, an increase in the F/C ratio was accompanied by a reduction in the O/C ratio (0.02 to 0.01) and a small anomalous increase in the N/C ratio. The FWHM of the F1s signal too showed an inversion from 2.17 eV (PTFE/H_2_/2012) to 2.0 eV (PTFE/H_2_/2011) as compared to 1.92 eV (for pristine PTFE). From the trends of the core level XPS signals, we can deduce that the plasma-induced surface changes degenerate over time but are not completely reversible over the time period studied. However, in order to study the ageing effects in more detail, further studies need to be carried out, taking into account the studies of Wilson *et al.* [27] and Werner *et al.* [26].

### 2.3. Up Scaling of H2 Plasma Treatment of PTFE Powders

PTFE is frequently used as a lubrication additive in many engineering thermoplastic compounds like polyamide or polyoxymethylene compounds for tribological applications such as bearings, gears or sliding elements. Therefore it is desirable to scale-up the powder quantity for possible commercial use. One PTFE sample (PTFE MAX H_2_/10 h) was treated with hydrogen as a process gas in a larger drum to obtain quantities up to 25 kg. The plasma process was operated at a microwave frequency of 2.45 GHz; however, a power output of 900 W and a pressure of 0.4 mbar were applied. In the large-scale device, the sample exhibited an F/C ratio of 1.76 *vs.* 1.86 for pristine PTFE. This small change in the F/C ratio is in contrast with the expected results, a higher energy output did not led to a greater depletion of fluorine. According to Pringle *et al.*, lower energy ions alter the surface of the PTFE polymer more readily; thus, a higher applied power output of 900 W would result in less defluorination [6]. Furthermore, the probability of the plasma species interacting with the PTFE particle was lower due to the larger volume of the drum of the large-scale device. We believe that to compensate for the higher quantities, multiple passes of the polymer powders can help in achieving uniform and higher levels of functionalization. Therefore, to achieve an optimal plasma treatment, the best ratio between the filling quantity, size of the device, treatment time and passes must be experimentally determined and further work is being carried out in this regard.

### 2.4. Fourier Transformed Infrared (FTIR) Spectroscopy

**Figure 5:**
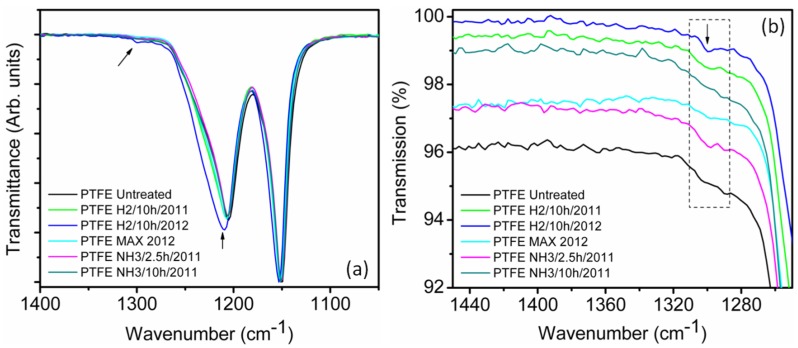
ATR FTIR spectrum of treated and pristine PTFE samples (**a**) In the region between 1250 cm^−1^ and 1450 cm^−1^ showing the slightly stronger reflection of the bands of PTFE H_2_/10 h and PTFE NH_3_/2.5 h in the vicinity of 1300 cm^−1^. (**b**) in the region between 1050 cm^−1^ and 1400 cm^−1^ showing the shifted bands of PTFE H_2_/10 h.

The FTIR spectra of pristine and plasma treated samples are shown in Figure 5a,b where the characteristic bands of CF_2_ wagging, CF_2_ stretching and CF_3_ stretching could be observed at 620–640 cm^−1^ (not shown here), 1150 cm^−1^ and 1240 cm^−1^, respectively [31]. The absorbance band observed at 1300 cm^−1^ in the spectra of the H_2_-treated sample (PTFE H_2_/10 h) and NH_3_-treated sample (PTFE NH_3_/2.5 h) indicated that slight changes occurred on the surface of the PTFE. In Figure 5a, the absorbance of hydrogen-treated PTFE (PTFE H_2_/10 h/2012) was located at 1210 cm^−1^, which was higher than the bands of the other samples at 1205 cm^−1^. The frequency and intensity shift of this band was assigned to the transformation of C–F bonds into C–H bonds and possibly C-CF_3_ bonds. The bands of all of the other treated samples were on the same level as the bands of pristine PTFE. According to Heitz *et al.*, for NH_3_-treated sample, the band at approx. 1300 cm^−1^ can be attributed to the formation of ammonium fluoride [32]. As described by Dorschner *et al.*, ammonium fluoride disappeared after post-treatment under vacuum at 100 °C [33]. In addition, for NH_3_ plasma treated samples, some weak and broad bands were observed in the range of 1550 and 1700 cm^−1^, which were absent in the pristine samples, for which the band assignment is ambiguous. The adsorbed water molecules are known to be broadly centered at 1600 cm^−1^, however they show a sharp spike which were not observed in our case [28]. Alternatively, these peaks could indicate the presence of amide with absorptions of C=O at 1637 cm^−1^ and NH/CN at 1578 cm^−1^, or attributed to N=O or C≡N, which are known to occur in this region [27].

### 2.5. Differential Scanning Calorimetry

**Figure 6:**
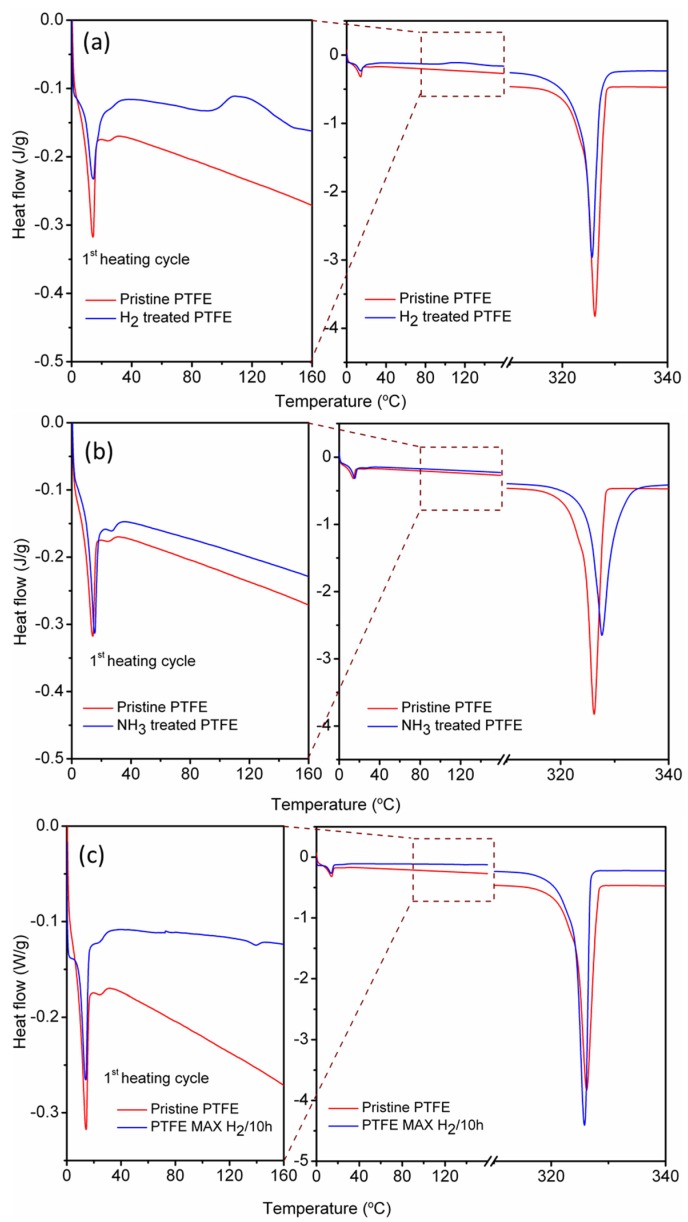
DSC curves of 1st heating cycles of (**a**) H_2_ plasma treated PTFE sample. (**b**) NH_3_ plasma treated PTFE sample. (**c**) MAX H_2_ plasma treated PTFE powders. The expanded curves on the left show the post-crystallization peaks observed for the plasma treated samples.

As seen in Table 2, no considerable difference was obtained in the melting temperatures of the pristine and plasma treated PTFE powders; however, the melting enthalpy showed considerable differences upon plasma treatment. It should be noted that the values reported here for the melting point and melting enthalpy are quite similar to those reported by Dumitras and Odochian [34]. The enthalpy calculated for pristine PTFE powders is ~66.0 J/g, giving a crystallinity value (ΔX_c_) of nearly 80.5% (with ΔH_m100_ for 100% crystalline PTFE being 82.0 J/g). Upon H_2_ plasma treatment, the ΔH_m1_ value reduces to 58.0 J/g providing a ΔX_c_ of 70.7% (Figure 6a). Similarly, upon NH_3_ plasma treatment the ΔH_m1_ value reduces to 62.0 J/g providing a ΔX_c_ of 75.6% (Figure 6b). However, samples PTFE MAX H_2_/10 h did not show any major changes in the ΔH_m1_ value and displayed a value quite close to that of pristine PTFE at 67 J/g providing a ΔX_c_ of 81.7% (Figure 6c). The DSC curve of PTFE H_2_/10 h/2012 and PTFE MAX H_2_/10 h showed signatures of post-crystallization in the range of 100–150 °C, which is again indicative of the changes in the chemical structure of treated polytetrafluoroethylene. In fact, the changes in the DSC curves correlate quite well with the XPS analysis in which high levels of defluorination were observed for the samples marked PTFE NH_3_/10 h/2011 (F/C ratio 1.13) and PTFE H_2_/10 h/2012 (F/C ratio of 1.30) as compared to pristine PTFE (F/C ratio 1.86). In the case of PTFE MAX H_2_/10 h, no significant defluorination was observed (F/C ratio of 1.76) manifesting itself into little observed change in the DSC scans. This reduction of crystallinity values for plasma treated samples can be explained by the collisions of the energetic gas phase particles and plasma species with the PTFE particles, leading to the bulk heating of the PTFE particles, bulk polymer chain relaxation and lowering of crystallinity.

**Table 2:** Summary of various differential scanning calorimetric values of different PTFE samples.

Sample		PTFE Pristine	PTFE H_2_/10 h/New	PTFE MAX H_2_/10 h	PTFE NH_3_/10 h
1st heating cycle					
T_m10_	[°C]	14	14	14	15
ΔH_m10_	[J/g]	8	7	7	8
T_k_	[°C]	–	116	133–167	–
ΔH_k_	[J/g]	–	6	9	–
T_m1_	[°C]	326	327	326	328
ΔH_m1_	[J/g]	66	58	67	62
ΔX_c_	[%]	80.5	70.7	81.7	75.6
2nd heating cycle					
T_m20_	[°C]	19/28	18/28	18/28	19/30
ΔH_m20_	[J/g]	11	8	11	8
T_m2_	[°C]	327	326	327	328
ΔH_m2_	[J/g]	65	57	67	54

### 2.6. Contact Angle Measurement (Washburn Capillary Rise Method)

**Figure 7:**
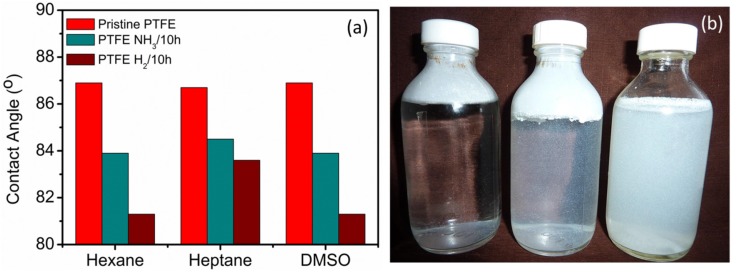
(**a**) Variation of contact angle for pristine and plasma treated samples in various testing media, (**b**) Pristine PTFE powders are poorly dispersible in water (**left**); upon H_2_ (**middle**) and NH_3_ plasma treatment (**right**), the dispersion is significantly improved.

Figure 7a shows the results of the contact angle measurement of pristine and plasma treated PTFE samples. The wettability measurements carried out for three different liquids with varying surface energies including Hexane (18.4 mJ/m^2^), Heptane (20.2 mJ/m^2^) and Dimethyl Sulfoxide, DMSO (43.6 mJ/m^2^). Between the NH_3_ and H_2_ plasma treated PTFE powders, even though the NH_3_ powders show much higher defluorination values as well as higher dispersion in water; the contact angle, as determined by Washburne capillary method, shows slightly lower values for H_2_ plasma treated powders. In accordance with the XPS results, the addition of polar moieties via plasma treatment led to a reduction in the contact angle. The plasma functionalization of PTFE powders leads to an increase in the surface free energy enhances the dispersion of the polymer powders via increase in the dispersive component. Park *et al.* [35] have reported that there is a correlation between the incorporation of functional groups and the atomic structure and hydrophilicity (wettability). The decrease of the contact angle is mainly due to the change in the surface concentration of the polar moieties. Increasing nitrogen/oxygen functionalities on plasma treated surfaces lead to a decrease in the contact angle of the powders so that surface becomes more hydrophilic. To better visualize the change in the surface functionality and its effect on the surface wettability, pristine and plasma treated powders were dispersed in water (0.1 g/10 mL). As observed clearly in Figure 7b, while, the pristine PTFE shows poor dispersion; the PTFE powders functionalised by H_2_ and NH_3_ plasmas display significantly better dispersion, which can be correlated to the presence of the polar groups.

## 3. Experimental Section

### 3.1. Materials and Plasma Functionalisation

Polytetrafluoroethylene powder samples (3M^™^ Dyneon^™^ PTFE Micropowder TF 9201Z, Burgkirchen, Germany) with a primary particle size of 200 nm and an average particle size of 6 μm were supplied by Sitraplas GmbH. The as-obtained PTFE powder samples were treated in a “Nano” plasma device (Diener electronic GmbH + Co. KG, Ebhausen, Germany) equipped with a rotary drum made of glass (Suppl. Figure S3), which is capable of performing plasma treatments on up to 3 kg of powder at a time. The plasma device is software controlled which enables a continuous regulation and documentation of all process relevant parameter. After the PTFE powders were placed into the glass drum, a vacuum between 0.2 mbar and 0.8 mbar, (Suppl. Table T2) was created using a rotary vacuum pump. Once the required pressure was achieved, the required process gas (H_2_ or NH_3_) was fed into the drum. The high voltage between the electrodes in the glass drum and suitable working pressures excites the plasma. The process was operated at a microwave frequency of 2.45 GHz and a power output of 270 W, except for the sample in the scale-up reactor drum, which was performed at 900 W. Due to the rotation of the glass drum, the PTFE powder particles pass the plasma excitation between the electrodes with the rotation and the gas flow rate held constant to ensure uniform treatment of powders. Fresh gas was continuously fed into the drum while the processed, contaminated gas was evacuated while maintaining the chamber pressure. Different exposure times (2.5 h and 10 h) were applied for NH_3_-treated samples to determine the impact of the treatment time. For the H_2_ plasma treated samples, the PTFE powders were only exposed for 10h to the plasma due to the low defluorination values observed for H_2_ plasma, as compared to NH_3_ plasma. One PTFE sample treated with hydrogen (PTFE H_2_/10 h/2011) was stored for 12 months in dark at ambient (25 ± 1 °C) temperature to investigate the degeneration of the plasma treatment and ageing effects. To scale-up the powder quantity for possible commercial use, one PTFE sample (PTFE MAX H_2_/10 h) was treated with hydrogen as a process gas in a larger device, Tetra 2400, to obtain quantities up to 25 kg. The plasma process was again operated at a microwave frequency of 2.45 GHz; however, a power output of 900 W and a pressure of 0.4 mbar were applied to accommodate for the larger sample size (Suppl. Table T2).

### 3.2. Characterisation Methods

Differential Scanning Calorimetry (DSC) data were generated using a DSCQ1000 (TA Instruments, Waters GmbH, Eschborn, Germany). The PTFE samples were sealed in aluminum crucibles and were heated from 0 °C to 350 °C in the first heating cycle. Subsequently, the samples were cooled to 0 °C, and a second heating cycle was applied at temperatures up to 350 °C. The measurements were performed in a dry N_2_ atmosphere at a flow rate of 50 mL/min. The heating and cooling rates were set to 10 °C·min^−1^. To generate Fourier transform infrared spectroscopy (FTIR) spectra, the PTFE samples were pressed into thin specimens and were measured on a Centaurus IR microscope coupled to a Nexus IR spectrometer (Thermo Electron Corporation, Thermo Fisher Scientific, Dreieich, Germany) using an ATR-device with Ge crystal. The number of scans was 64 with the resolution set at ±2 cm^−1^. The X-ray photoelectron spectroscopy (XPS) spectra were collected with an AXIS Nova Spectrometer (Kratos Analytical Ltd., Manchester, UK) using a monochromated AlKα X-ray source (excitation energy of 1486.6 eV). The samples were mounted using high-vacuum carbon tape and the powders were pressed onto the tape using clean glass slides to provide a smooth surface for analysis. For energy calibration, the samples were energy calibrated to the (–CF_2_-)_n_ peak in the high-resolution C1s spectra at 292.8 eV or the F1s signal at 689 eV. To analyze the chemical bonding state of atoms, high-resolution C1s spectra were deconvoluted into Gaussian functions, fitting the experimental curves using a nonlinear, least-squares program (Casa XPS). The relative abundance of chemical groups corresponding to a set of deconvoluted peaks was estimated from their relative peak areas calculated using a linear background correction. Thermal stability XPS analysis of the samples was carried out by mounting the powder samples on Beryllium Copper (BeCu) films. The low thickness of the BeCu films in conjunction with their high thermal conductivity ensures excellent heat transfer for thermal analysis. The thermocouple to measure the sample temperature was mounted on top of the sample surface. The temperature dependent XPS measurement were carried out at temperatures of 30 °C, 100 °C, 200 °C, 300 °C and 325 °C (melting point of the PTFE) by heating the samples inside the XPS chamber under UHV conditions. The XPS spectra were taken once the samples had cooled back down to the room temperature after the heating cycle. Contact angle of the samples were measured using the Washburn method [24] using the KRUSS Tensiometer K100 (KRUSS GmbH, Hamburg, Germany). The method is employed to measure the contact angle and the surface free energy of the porous substances such as bulk powder or pigments, and absorbent materials. The powder (about 1 g) to be measured is filled into a cylindrical tube with a filter base and suspended from the balance. After the vessel has contacted the liquid the speed at which the liquid rises through the bulk powder is measured by recording the increase in weight as a function of time. Bulk powder through which a liquid flows can be regarded as being a bundle of capillaries. This means that for the calculation of the advancing angle, which corresponds to the contact angle between the solid and the liquid, the following Washburn equation, which applies to capillaries, can be used:

m^2^/t = c. ρ ^2^.σ.cos θ/η (1)

where m is mass; t is flow time; σ is the surface tension of the liquid; c is capillary constant of the powder; ρ is the density of the liquid; θ is the contact angle and η is the viscosity of the liquid. The constant *c* includes the number of micro-capillaries and their mean radius, and depends on the nature of the powder and also on that of the measuring tube. Plotting the square of the mass m [20] against time t shows a linear region, the slope of which, for known liquid properties (σ, ρ and η), only contains the two unknowns c and *θ*. To determine the constant c, a measurement is carried out with an optimally spreading liquid, a non-polar liquid (e.g., n-hexane), with which the contact angle *θ* is 0° (*cos θ* = 1). The value of c is substituted in the equation in order to determine the contact angle *θ* with the help of other liquids. The contact angle measured in this way is an advancing angle, as it is measured in the course of wetting. As c depends on the bulk density, it must be ensured that the powder is packed consistently for all measurements on the same powder. Contact angles greater than 90° cannot be measured using this method, as no wetting of the powder takes place.

## 4. Conclusions

The PTFE powders were treated with 2.45 GHz H_2_ and NH_3_ microwave plasmas to functionalize and chemically modify their surface; which were further characterized by wettability measurements, XPS, FTIR and DSC analysis. The XPS results reveal that as compared to H_2_ plasma treatment, the NH_3_ plasma treatment was much more effective in defluorination of PTFE and the resulting F/C ratio was directly proportional to the treatment time, with higher treatment times reducing the F/C ratio significantly and enhancing the wettability and dispersion of PTFE powder in water. The effects of plasma treatment could also be observed clearly in the DSC scans, where a higher defluorination value led to a lower crystallinity of the samples. To assess the effects of long term ambient storage on the plasma induced functionalities, H_2_ plasma treated powder was stored in ambient storage conditions for a period of 12 months. From the trends observed in the core level XPS signals, it was deduced that the plasma induced surface changes degenerate over time, however, in the time period studied, they were not completely reversible. Through temperature dependent XPS analysis of pristine and plasma treated PTFE, the removal of hydrogen and nitrogen functional groups from PTFE surface was observed, especially above 200 °C. However, a complete removal of the moieties was not observed even at high temperatures (>300 °C), thus indicating at the stability of the functional groups on the PTFE surfaces. Furthermore, to study the feasibility of scaling up the plasma treatment process a scale-up plasma treatment reactor with a larger drum was designed. Initial results indicated lower defluorination values signifying that further optimization of processing conditions is required. The measurement of wettability proved the contact angles decreased upon plasma treatment, thus confirming that the microwave plasma functionalization of PTFE powders is an effective method of chemical modification.

## Supplementary Materials

Supplementary materials can be accessed at: http://www.mdpi.com/1996-1944/8/5/2258/s1.
