# Supplementary material for: Low-Pressure H2, NH3 Microwave Plasma Treatment of Polytetrafluoroethylene (PTFE) Powders: Chemical, Thermal and Wettability Analysis
Source: Materials (Basel). 2015 Apr 28;8(5):2258–75. doi: 10.3390/ma8052258 (PMC5455540; doi:10.3390/ma8052258)
Supplement: Supplementary file 1 [file materials-08-02258-s001.pdf]

Supplementary Information

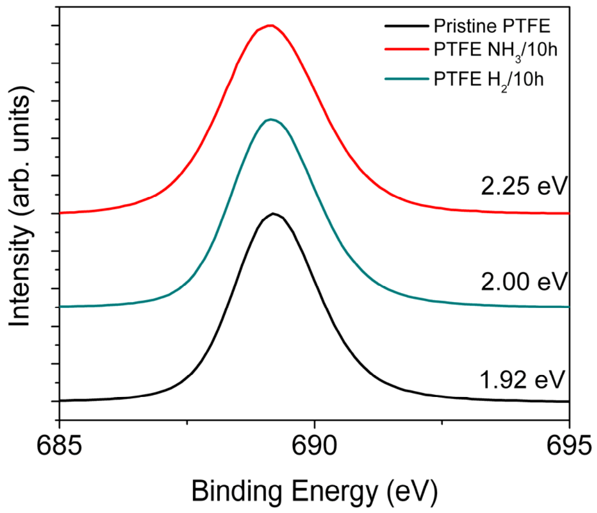

Figure S1. XPS F1s spectra for pristine and plasma treated PTFE powders showing the increase in the F1s FWHM.

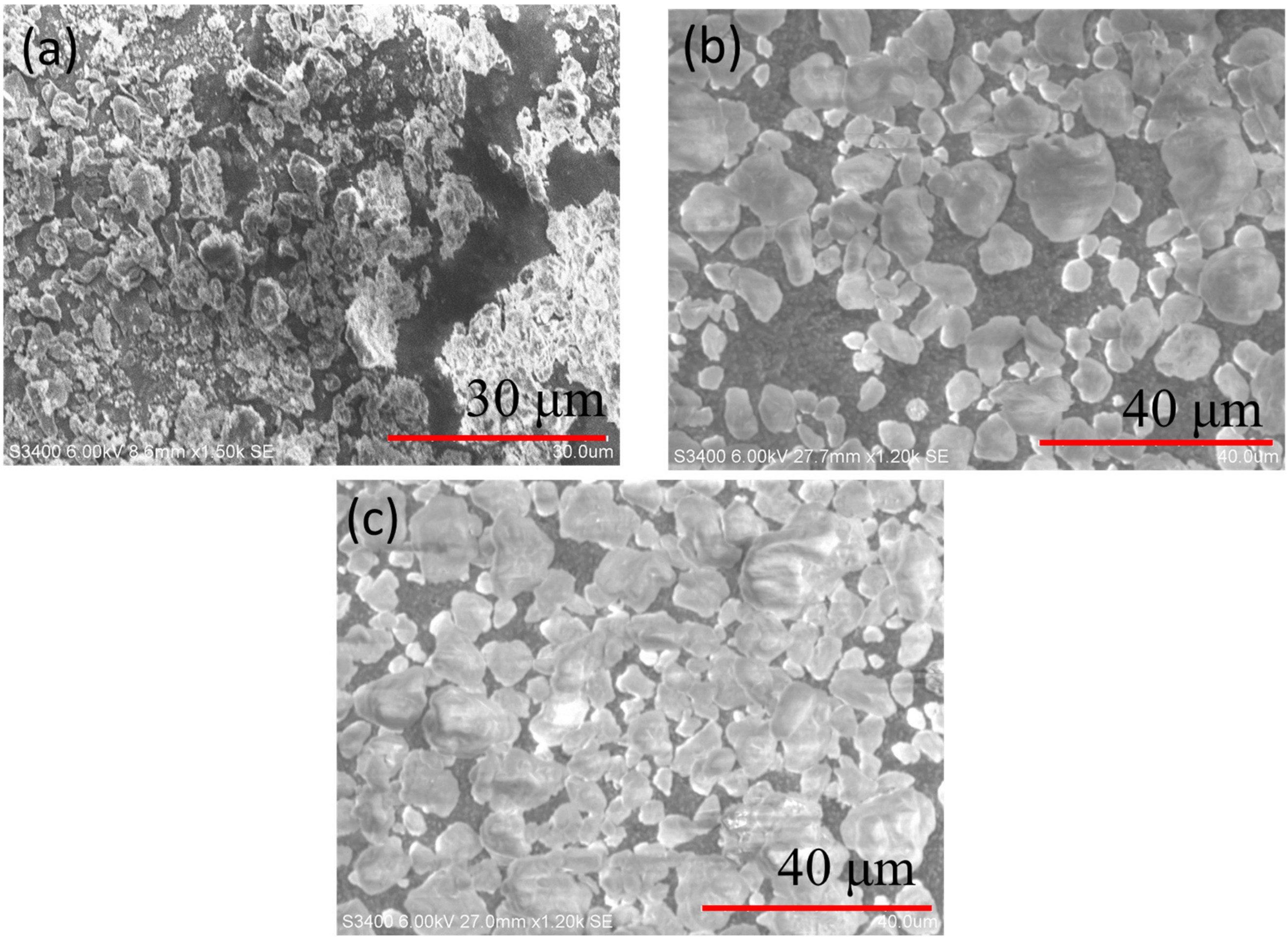

Figure S2. SEM images of (**a**) pristine, (**b**) H_2-plasma treated and (**c**) NH_3-plasma treated PTFE powders.

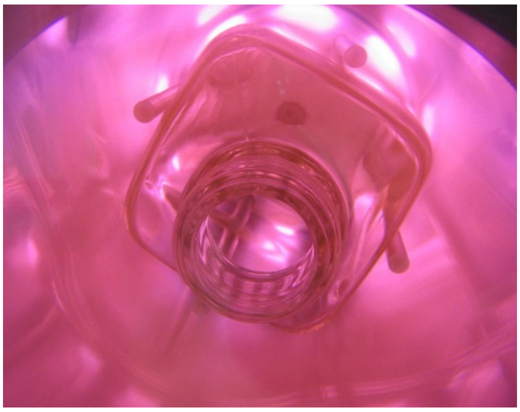

Figure S3. Excitation of the microwave plasma inside the glass drum of the “nano“ plasma device.

Table T1. Atomic percentages and associated elemental ratios of PTFE samples at various temperatures.

Sample Temperature	Atomic Percentage [%]				Elemental Ratios		
**PTFE NH_3/10 h**	**C**	**F**	**O**	**N**	**F/C**	**O/C**	**N/C**
30 °C	44.85	50.81	1.66	2.68	1.13	0.04	0.06
100 °C	47.48	47.51	1.94	3.07	1.00	0.04	0.06
200 °C	44.75	52.19	1.03	2.04	1.16	0.02	0.04
300 °C	43.36	54.97	0.49	1.18	1.26	0.01	0.03
325 °C	43.58	54.59	0.58	1.24	1.26	0.01	0.03
**PTFE H_2/10 h**							
30 °C	43.11	56.04	0.85	--	1.30	0.02	--
100 °C	46.88	52.39	0.73	--	1.12	0.01	--
200 °C	45.03	54.52	0.46	--	1.21	0.01	--
300 °C	44.09	55.59	0.32	--	1.26	0.01	--
325 °C	44.46	55.39	0.15	--	1.26	0.01	--

Table T2. Plasma samples and treatment parameters.

Sample Name	Process Gas	Effective Power [W]	Pressure [mbar]	Treatment Time [h]
PTFE NH_3/2.5 h	Ammonia	270	0.8	2.5
PTFE NH_3/10 h	Ammonia	270	0.8	10
PTFE H_2/10 h/2012	Hydrogen	270	0.8	10
PTFE H_2/10 h/2011	Hydrogen	270	0.8	10
PTFE MAX H_2/10 h	Hydrogen	900	0.4	10
